# Increased varus deformity requires greater personalisation of alignment: A finite element biomechanical study

**DOI:** 10.1002/jeo2.70482

**Published:** 2025-10-31

**Authors:** Arcangelo Russo, Mattia Sisella, Anna Carrara, Nicol Giacoppo, Giuseppe Gianluca Costa, Gianluca Zocco, Bernardo Innocenti

**Affiliations:** ^1^ Department of Medicine and Surgery Kore University of Enna Enna Italy; ^2^ Orthopaedic and Traumatologic Unit, Umberto I Hospital Azienda Sanitaria Provinciale di Enna Enna Italy; ^3^ BEAMS Department (Bio Electro and Mechanical Systems), École Polytechnique de Bruxelles Université Libre de Bruxelles Bruxelles Belgium

**Keywords:** inverse kinematic alignment, kinematic alignment, knee biomechanics, mechanical alignment, total knee arthroplasty

## Abstract

**Purpose:**

This finite element study aims to compare the biomechanical performance of three total knee arthroplasty (TKA) alignment strategies—mechanical alignment, kinematic alignment and inverse kinematic alignment—in knees with different degrees of varus deformity. We hypothesise that alignment choice has limited impact in rather neutral alignment but significant biomechanical effects in mild deformity.

**Methods:**

Two osteoarthritic patients scheduled for TKA, one with almost neutral alignment (1.5°) and one with mild (10.4°) varus deformity, were selected. For each patient, mechanical alignment, kinematic alignment and inverse kinematic alignment strategies were virtually applied using a posterior‐stabilised fixed‐bearing implant. Patient‐specific knee joint geometries, including bones and ligaments, were modelled from CT scans. Gait simulations were conducted following ISO standard. Outputs analysed and compared among alignments included contact areas and contact forces, von Mises stress on the tibial insert, tibial anterior‐posterior translation and tibial internal‐external rotation.

**Results:**

In the almost neutral knee, all three alignments showed comparable kinematics and kinetics. However, in the mildly deformed case, notable differences emerged. Kinematic alignment achieved more balanced medial‐lateral contact areas and forces, while inverse kinematic alignment displayed increased internal‐external rotation, indicating potential instability from suboptimal ligament balance. Mechanical alignment resulted in higher medial stress, while kinematic alignment redistributed stress more evenly. Inverse kinematic alignment exhibited intermediate behaviour, resembling mechanical alignment in force distribution under mild deformity.

**Conclusion:**

Alignment strategy plays a critical role in TKA biomechanics, especially in case of patients with mild varus deformity. While similar outcomes are achievable with almost no deformities, patient‐specific morphology heavily influences performance under more complex geometries. These findings support the adoption of a personalised alignment approach tailored to individual anatomical characteristics rather than rigid adherence to a single alignment philosophy.

**Level of Evidence:**

N/A.

**Abbreviations**
aMCL: anterior medial collateral ligament
AP: antero‐posterior
CoCrMo: cobalt‐chromium‐molybdenum alloy
CT: computed tomography
*FB*
: fixed‐bearing
FEA: finite element analysis
FJL: femoral joint line
GC: gait cycle
HKA: hip‐knee‐ankle angle
IE: internal‐external
iKA: inverse kinematic alignment
KA: kinematic alignment
LCL: lateral collateral ligament
MA: mechanical alignment
MCL: medial collateral ligament
OA: osteoarthritis
P1: patient 1
P2: patient 2
pMCL: posterior medial collateral ligament
PS: posterior‐stabilised
PTS: posterior tibial slope
Ti6Al4V: titanium‐aluminium‐vanadium alloy
TJL: tibial joint line
TKA: total knee arthroplasty
UHMWPE: ultra‐high‐molecular‐weight‐polyethylene

## INTRODUCTION

Limb alignment after total knee arthroplasty (TKA) has become one of the most discussed topics in the last few years. The mechanical alignment (MA) has been constantly supported by most physicians because of satisfactory long‐term survival rates [7]. However, this ‘systematic approach’ does not consider the anatomical peculiarities of each patient, including soft tissue balancing and alignment. Recent studies demonstrated neutral limb alignment is less common than expected, even in the healthy population [1, 8]. As a result, several alternative approaches were introduced, with the goal of reproducing native patients' alignment and improving clinical outcomes.

Kinematic alignment (KA) aims to reform the prearthritic joint line along the three knee kinematic axes, thus providing a more patient‐specific approach [9]. Bone resections are performed at the same thickness as the prosthetic components, after adjusting for cartilage wear. The major drawback appears in the case of patients with mild varus or valgus deformities, resulting in an extreme alignment that could theoretically impair implant survivorship. An additional risk is tibial over‐resection, which may occur due to gap mismatches and ligament balancing complications. The inverse kinematic alignment (iKA) technique has been introduced to overcome such problems [39]. Different from the KA technique, this tibia‐based technique aims to ‘resurface’ the tibia, maintaining the native tibial joint line (TJL) obliquity. Flexion and extension gaps are balanced by performing ligaments balancing and consequently adjusting the femoral resections. Despite promising early clinical results [40, 41], theoretical drawbacks of these alternative approaches include altered knee kinematics and asymmetrical load distribution, especially in mild deformities, which could impair implant survivorship. The clinical evidence about this issue is still sparse.

Accurate analysis of stress distribution and joint kinematics in prosthetic knees with different alignment strategies may help to improve the understanding of this important aspect. Finite element analysis (FEA) has been demonstrated as a valid tool to simulate several configurations [42], thus offering the possibility of evaluating those aspects that cannot be adequately investigated in vivo or even ex vivo.

The purpose of this FEA is to evaluate knee loads distribution and knee kinematics with three different alignment techniques (MA, KA and iKA), by simulating two clinical scenarios with diverse grades of deformity. The hypothesis is the three evaluated alignment approaches result in different outcomes, especially in the case of mild deformity.

## MATERIALS AND METHODS

The methodology adopted in the present study follows a commonly used and validated approach, that has been adopted in several biomechanical finite element studies [2, 12, 35].

### Geometries

Two patients with severe knee osteoarthritis (OA) who were candidates for TKA were selected. Both patients underwent standard preoperative radiographic evaluation including weight‐bearing full‐length antero‐posterior (AP) radiographs and weightbearing knee radiographs in three views (AP, lateral and Merchant view). The angle between the mechanical axis of the femur and the mechanical axis of the tibia was defined as mechanical tibio‐femoral angle and used to quantify the limb deformity, as well as the orientation of the femoral and TJLs. Furthermore, both patients underwent computed tomography (CT) scans for the purpose of this study. Informed consent was obtained from both participants for execution and for anonymous data acquisition.

The first patient (P1), a 76‐year‐old man with left knee OA, presented a rather neutral alignment of 1.5° varus (medial proximal tibial angle [MPTA] = 87°, lateral distal femoral angle [LDFA] = 87°). The second patient (P2), a 60‐year‐old woman with left knee OA, presented a mild varus deformity of 10.4° (MPTA = 85°, LDFA = 90°). Neither previous surgery (arthroscopy, ligament reconstruction or osteotomy) nor rheumatic disease were reported in the medical history of either patient.

The CT images were imported into an image processing software (3D Slicer, https://www.slicer.org/) and segmented to generate 3D femoral, tibial and fibular bone geometries of the two left knees. The anatomical bone landmarks were identified in the 3D environment [38] to determine and confirm the previously calculated mechanical and anatomical axes of femur and tibia, together with the trans‐epicondylar axis of the femur. The locations of the main soft tissue insertions were determined based on anatomical landmarks extracted from patient‐specific imaging data [38]. The medial collateral ligament (MCL) was modelled distinguishing two bundles, the anterior (aMCL) and posterior (pMCL) bundles, according to previous studies in the literature [13, 15, 31, 35]. For both patients, the fibula was used only for determining the insertion of the lateral collateral ligament (LCL), but it was not incorporated into the final knee models as 3D geometry [31]. Moreover, according to previous similar studies, patella was not included in the models, given its minimal influence on the investigated motor task [2, 13, 34]. Consequently, patellar tracking was not investigated in this study to reduce the computational time of the model.

### Model configurations

For each knee model, three configurations were developed to reproduce the three different alignment conditions: MA, KA and iKA. According to the surgical procedure of each TKA strategy, the required bone cuts were performed using an Anterior Reference surgical technique, in agreement with the senior surgeon's indications (A.R.). Specifically, five cuts were performed on the femur and one on the tibia. For the MA model, distal femoral cut and proximal tibial cut were achieved using cutting planes perpendicular to the mechanical axis of the respective bone. For the KA model, the femoral joint line (FJL) was identified in M/L and A/P directions, and replicated on the model, after applying a 2 mm adjustment to compensate for cartilage wear [24]. The initial cut was made on the distal femur, followed by the cut on the proximal tibia, again following the FJL. For the iKA model, the TJL was identified with the same approach. Then, the proximal tibial cut was performed following the TJL and the posterior tibial slope (PTS). Finally, the distal femoral cut was performed, also following the TJL. The management of the collateral ligaments was a key aspect of model development, reflecting its critical importance in clinical practice. To ensure comparability of results, in the model representing KA alignment, which conceptually does not imply any alteration of the patient's native ligaments, a physiological ligament stiffness derived from the literature was assigned. Based on this assumption, both ligament stiffness and prestrain were optimised to define the initial reference condition (as explained in the next section), following a validated FEA process. This initial step was justified by the fact that the two patients did not present any specific deficiency in their collateral ligaments. In the MA model, where ligament release is often required (especially with mild and severe deformities), these parameters were iteratively adjusted to reproduce ligament stresses comparable to those in the KA model. In the iKA model, where ligament balancing dictates the femoral cuts, the process was reversed: femoral resections were iteratively optimised to reproduce ligament stresses comparable to the KA model. This approach allowed us to evaluate the performance of the three implants with appropriate ligament balancing, induced by different cuts and alignments. Each in silico model generated through the computational workflow was reviewed and approved by the senior surgeon (A.R.) prior to the analysis. This ensured consistency with surgical practice and alignment with clinical experience.

For all the configurations, a cementless genus posterior‐stabilised (PS) fixed‐bearing (FB) total knee prosthesis (Adler Ortho) was virtually implanted. According to the dimensions of the bones of the two patients, the following prosthesis sizes were selected: size 7 with a 12 mm thickness insert for P1, and size 4 with a 14 mm thickness insert for P2.

### Material model and properties

For all materials involved in this study, homogeneous, linear‐elastic models were adopted, in line with previous studies in literature [3, 13, 16, 35]. This assumption provides a reasonable approximation of the mechanical properties, enabling a qualitative comparison across different configurations [14]. Cancellous bone was modelled as an isotropic material, while cortical bone was modelled as a transversely isotropic material [3, 6, 12]. Femoral, tibial and insert components were considered in cobalt‐chromium‐molybdenum alloy (CoCrMo), titanium‐aluminium‐vanadium alloy (Ti6Al4V) and ultra‐high‐molecular‐weight‐polyethylene (UHMWPE), respectively, all assumed to behave isotropically [3, 11, 14, 16]. The ligaments were modelled as linear elastic isotropic materials, represented as one‐dimensional elements connected to the centroid of each insertion area. They were characterised by a defined circular cross‐section and prescribed prestrain [15], which was optimised through an iterative process. The initial strains of the LCL and of the two bundles of the MCL were derived from the literature [13, 14, 15]. A coefficient of friction of 0.04 was applied between the femoral component and the tibial insert, and a coefficient of friction of 0.4 between the tibial plate and the tibial bone [12, 13, 14, 36]. A complete overview of the material models and properties used in the study is provided in Table 1.

**Table 1:** Material model and properties of the knee models.

Material	Material behaviour	Young's modulus (MPa)	Poisson's ratio	Initial strain ε_r_
Cortical bone	Transversely Isotropic	E_1_ = 17,000; E_2_ = 11,500; E_3_ = 11,500;	ν_12_ = 0.31; ν_13_ = 0.31; ν_23_ = 0.51;	/
Cancellous bone	Elastic Isotropic	2130	0.31	/
Cobalt‐chromium‐molybdenum alloy	Elastic Isotropic	240,000	0.3	/
Titanium‐aluminium‐vanadium alloy	Elastic Isotropic	114,000	0.3	/
Ultra‐high‐molecular‐weight‐polyethylene	Elastic Isotropic	724.2	0.46	/
Anterior medial collateral ligament	Elastic Isotropic	196	0.45	0.04
Posterior medial collateral ligament	Elastic Isotropic	196	0.45	0.03
Lateral collateral ligament	Elastic Isotropic	111	0.45	0.05

*Note*: For the cortical bone, the direction E_1_ represents the longitudinal direction and corresponds to the anatomical axis of the bone.

### FEA

FEA was conducted using the software Abaqus/CAE 2019 (Dassault Systémes, Vélizy‐Villacoublay). Each configuration was analysed during a dynamically simulated gait cycle (GC), which was replicated by applying appropriate boundary conditions (including flexion‐extension rotation, AP force, axial force and rotational torque) according to the ISO‐14243‐1:2009 [19].

For each model, linear tetrahedral elements (C3D4 elements) were used for all parts, except for the ligaments, which were meshed using linear line elements (B31). An approximate global element size of 3 mm was assigned to the femur, tibia, femoral and tibial components. However, to improve reliability, a global element size of 2 mm was applied for the insert, while element sizes of 1 and 0.5 mm were chosen for the ligaments of P1 and P2, respectively. Meshing quality was verified through convergence analysis [32].

For each model, tibio‐femoral kinetics and kinematics were extracted and compared among the configurations. In particular, medial and lateral contact areas on the insert, medial and lateral forces due to contact pressure, von Mises stress on the insert, tibial AP translation and tibial internal‐external (IE) rotation were evaluated.

## RESULTS

**Figure 1:**
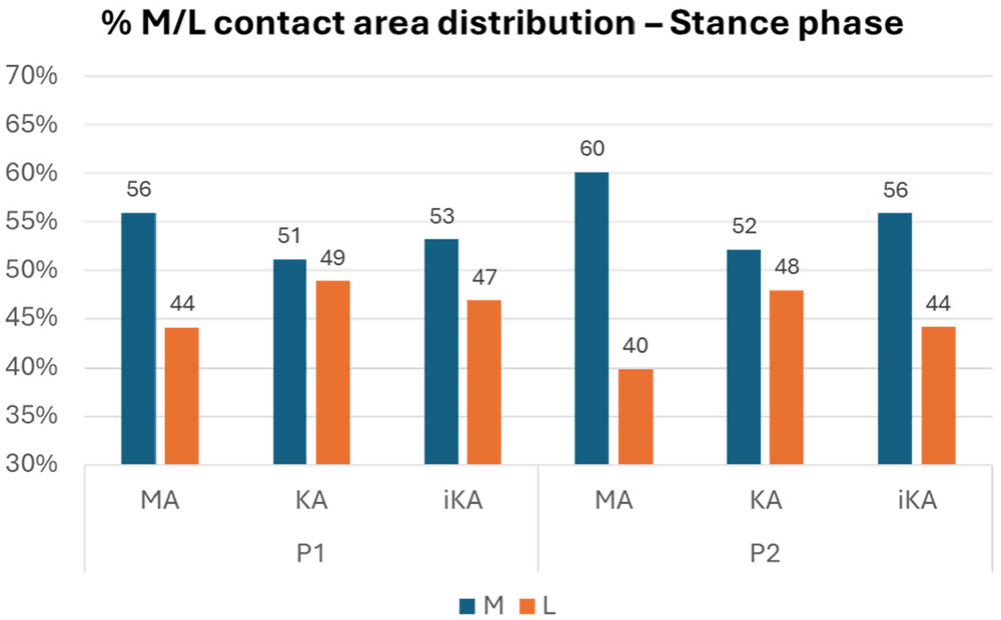
Percentual M/L contact area distribution during stance phase in mechanical alignment (MA), kinematic alignment (KA) and inverse kinematic alignment (iKA) models for P1 and P2.

Medial and lateral average contact areas are significantly influenced by the degree of varus deformity across the three alignment strategies, with intermodel differences becoming more pronounced as deformity increases. In both patients, the differences between medial and lateral compartments are more pronounced in the MA model. The KA model, in contrast, shows more similar values between the lateral and medial contact areas. The iKA model demonstrates an intermediate pattern, achieving a more balanced distribution than the MA model but not as evenly distributed as the KA model (Figure 1). As expected, for all models and for both patients, average contact area values are lower during the swing phase compared to the stance phase, and consistently higher in the medial compartment. Tables 2 and 3 show the average values of medial and lateral contact areas during stance and swing phase for P1 and P2.

**Table 2:** Average lateral and medial contact areas during stance phases in mechanical alignment (MA), kinematic alignment (KA) and inverse kinematic alignment (iKA) models for P1 and P2.

	P1		P2	
	Lateral (mm^2^)	Medial (mm^2^)	Lateral (mm^2^)	Medial (mm^2^)
MA	11	149	95	142
KA	131	132	96	105
iKA	126	141	68	87

**Table 3:** Average lateral and medial contact areas during swing phases in mechanical alignment (MA), kinematic alignment (KA) and inverse kinematic alignment (iKA) models for P1 and P2.

	P1		P2	
	Lateral (mm^2^)	Medial (mm^2^)	Lateral (mm^2^)	Medial (mm^2^)
MA	82	129	98	169
KA	100	119	107	112
iKA	88	139	73	112

For both patients, the three models show similar outputs when analysing the differences in stance and swing contact force patterns. During the stance phase, there is a notable increase in force compared to the swing phase, which accounts for the higher contact area values observed during stance. For Patient 1, across all models and throughout the entire motion, higher force values are observed on the medial side of the insert compared to the lateral side. During the swing phase, the KA model shows a slight force redistribution at peak flexion, with an average contact force of 734 N in the lateral compartment and 1133 N in the medial compartment. For Patient 2, the MA model produces outputs comparable to those of Patient 1. However, in the KA and iKA models, the medial and lateral contact forces are generally more balanced, even though the M/L ratio is inverted between KA and iKA. During the stance phase, the average contact force is 1872 N in the lateral compartment and 1618 N in the medial side, while during swing, it is 1166 N in the lateral compartment and 958 N in the medial compartment. In contrast, the iKA model exhibits greater instability and lower force values, with an average contact force of 1130 N in the lateral compartment and 1184 N in the medial side during the stance phase.

**Figure 2:**
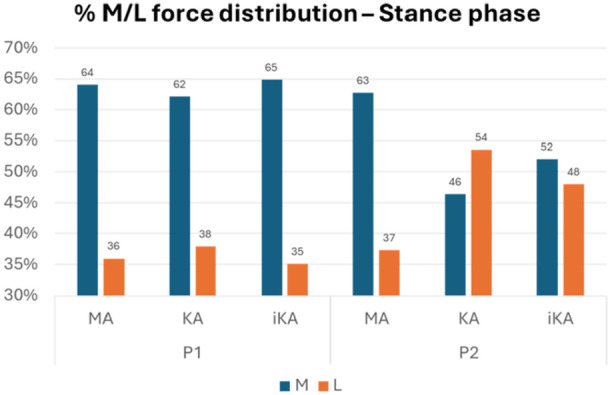
Percentual M/L force distribution during stance phase in mechanical alignment (MA), kinematic alignment (KA) and inverse kinematic alignment (iKA) models for P1 and P2.

Regarding force distribution, the results of the three alignment strategies depend on the patient deformity (Figure 2). For P1 with almost neutral alignment, the differences between the three alignments are minimal, with an M/L ratio of around 60%‐40%. For P2, when the varus deformity is mild, MA returns an equal M/L ratio compared to P1. On contrary, KA and iKA generates a force distribution that is different from 60%–40%, and equal to 50%–50%.

**Figure 3:**
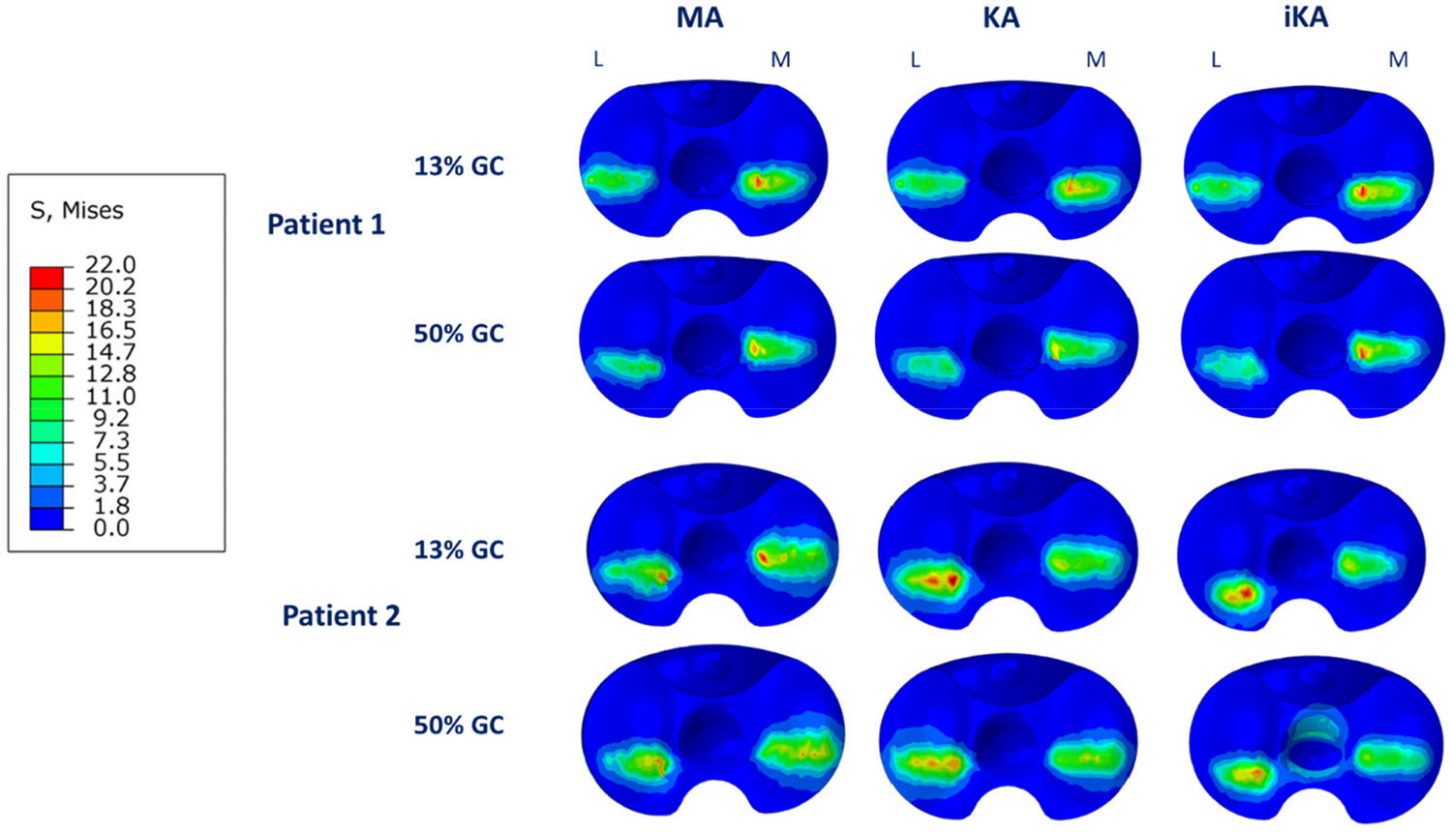
von Mises stresses on the insert in different instants of the gait cycle (GC) in mechanical alignment (MA), kinematic alignment (KA) and inverse kinematic alignment (iKA) models for P1 and P2 (values shown in MPa).

The contact area and the stress distribution are typically concentrated on the superior medio‐lateral axis, with a tendency to be more posterior (Figure 3). The stress distribution is not symmetrical between the two compartments, generally higher in the medial compartment due to the higher force. Also, for UHMWPE stress, no substantial differences are observed for Patient 1. However, the KA model shows slightly lower stress values, more evenly distributed between the medial and lateral compartments compared to the MA model. Specifically, for Patient 1, the maximum von Mises stress in the KA model is 19.9 MPa at 13% GC, compared to the 21.1 MPa in the MA model and 21.8 MPa in the iKA model. At 50% GC, the KA model shows a maximum stress value of 16.7 MPa, while the MA and iKA models exhibit higher values of 18.2 and 21.3 MPa, respectively. In contrast, for Patient 2, the KA model produces a change in stress and contact pressure, decreasing stress in the medial compartment and increasing it in the lateral compartment. The iKA model exhibits a highly asymmetric stress distribution between the medial and lateral regions.

**Figure 4:**
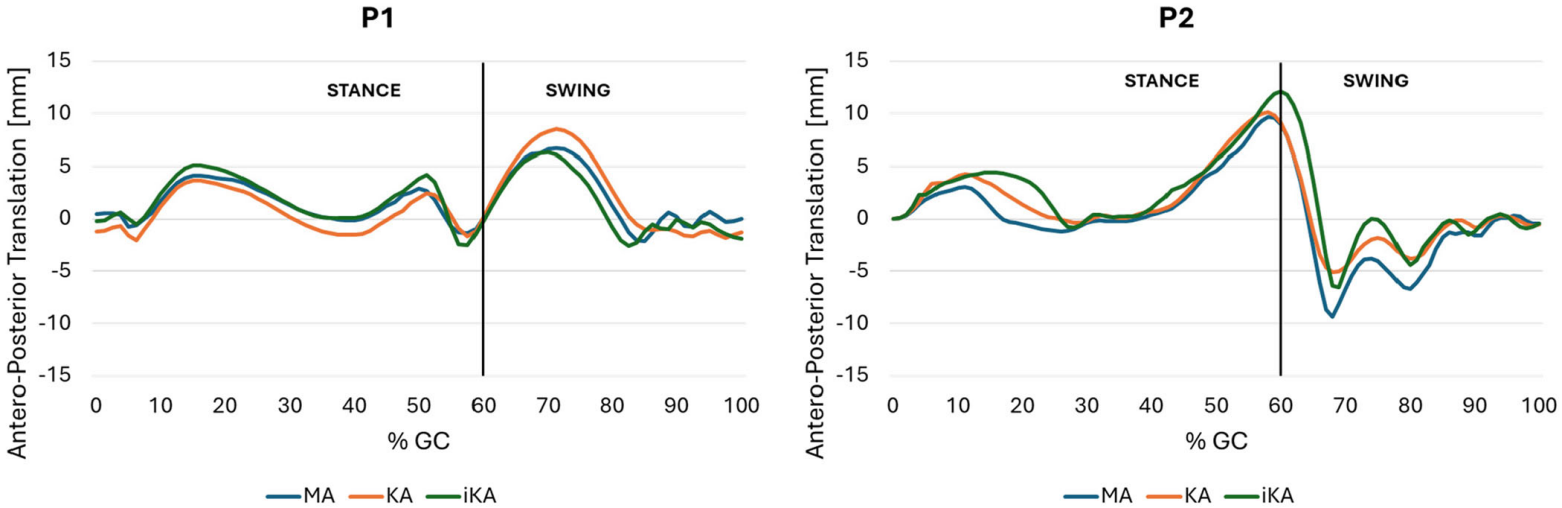
Tibial Antero(+)‐Posterior(‐) translation during the gait cycle in mechanical alignment (MA), kinematic alignment (KA) and inverse kinematic alignment (iKA) models for P1 and P2. GC, gait cycle.

Focusing on tibial AP tibial translations, the three alignment strategies exhibit generally similar patterns for each patient, with differences observed at key instants of the gait cycle, as well as interpatient variability (Figure 4). Nonetheless, intrapatient variability remains minimal. Specifically, two notable translations occur at distinct phases of the cycle. Both patients show an anterior displacement of approximately 5 mm during the stance phase. However, during the swing phase, P2 exhibits an anterior translation exceeding 10 mm, whereas P1 remains within the stance‐phase range.

**Figure 5:**
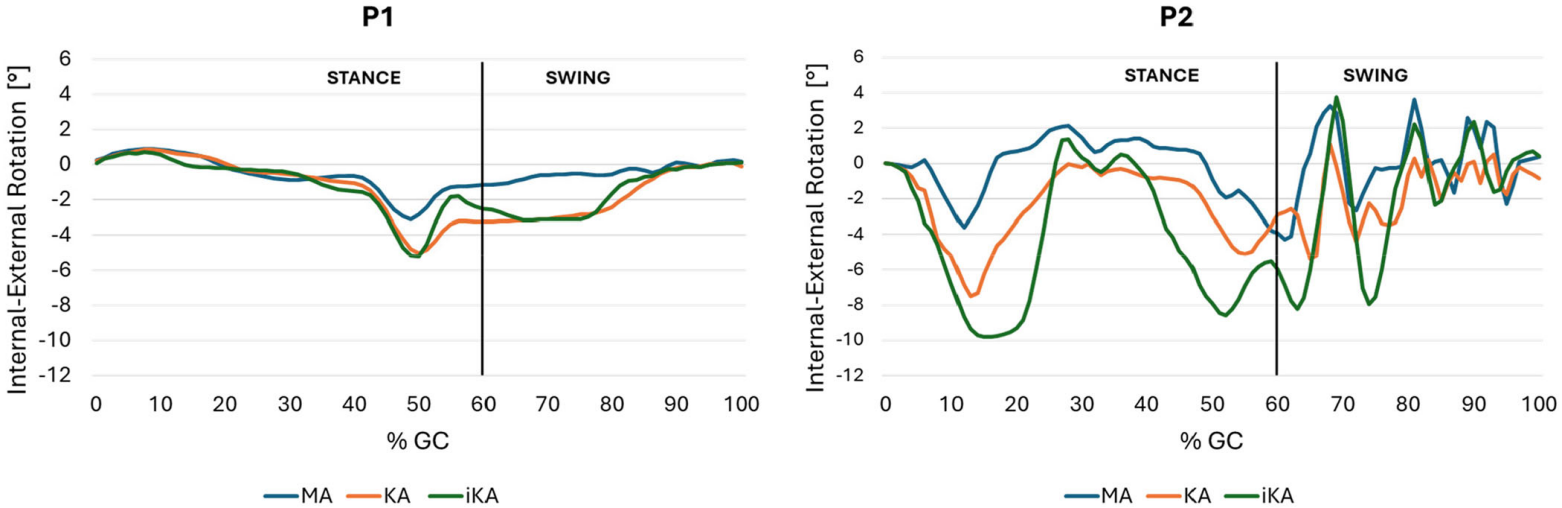
Tibial Internal(‐)‐External(+) rotation angles during the gait cycle in mechanical alignment (MA), kinematic alignment (KA) and inverse kinematic alignment (iKA) models for P1 and P2. GC, gait cycle.

Regarding IE rotation (Figure 5), the main differences between alignments strategies for P1 occur during the swing phase and these are limited to approximately 2° of external rotation. For P2, the IE rotation range is broader, and the differences between alignment strategies are more pronounced. In particular, at 15% of the gait cycle, when the maximal force is applied, external rotation increases to 10° in the iKA model.

## DISCUSSION

The main finding of the present study is that each TKA alignment strategy generates a different impact on the biomechanical behaviour of the prosthetic knee, which is significantly influenced by the severity of the varus deformity. Specifically, this study evaluated different biomechanical parameters, such as tibiofemoral contact areas and stress distribution, which may influence knee function and implant longevity.

The results of the present study are consistent with values reported in the literature, generally reported as 55%–45% between medial and lateral compartments [14, 40], although some studies suggest they may be closer to 60%–40% [7, 13, 28]. The similarity in contact force values observed in P1 among the three alignment strategies is likely explained by the almost neutral alignment, which results in comparable bone cut orientations across the different alignment strategies. It is noteworthy that KA exhibits the most symmetrical stress distribution in this context, reflecting the preservation of the knee's preoperative morphology and a more balanced contact. In contrast, the differences between the three alignment strategies in P2 become more pronounced, as the mild varus deformity of 10.4° leads to greater variations in bone cut orientation. MA fully restores the 60%–40% force distribution again. This implies that MA maintains a standard force distribution, regardless of the initial patient geometry. Conversely, in both the KA and iKA models, a more symmetric force distribution between medial and lateral tibiofemoral compartments was found. Interestingly, KA resulted in an inverted force distribution in the patient with more pronounced varus deformity, resulting in increased forces on the lateral compartment in the stance phase despite a nearly symmetrical contact area distribution.

In the literature, the impact of alignment strategies on tibiofemoral load distributions has been extensively evaluated in recent years, producing conflicting results. Nakamura et al. [27] compared MA and KA kinematics in four bone models with increasing varus deformity. In the severe varus case, the load on the medial compartment in the KA model was found to increase between 32.2% and 53.7% when compared to MA. Similarly, Ishikawa et al. [18] reported increased tibiofemoral contact stresses in KA TKA using a musculoskeletal computer simulation. Song et al. [37] recently compared MA and KA strategies in FEA of a medial pivoting knee design. Although KA achieved closer‐to‐normal knee biomechanics by allowing more posterior translation of the lateral tibial plateau, the authors discovered higher contact stresses in KA than in MA. Conversely, Kang et al. [20] found improved kinematics of KA over MA, with reduced contact pressure due to the increased contact area, as observed in the present study. A more recent FEA conducted on 10 different knee phenotypes corroborated such findings [21], reporting reduced or comparable contact pressures on the polyethylene bearing surface through increased or maintained contact area over a gait cycle. In confirmation of this, two recent systematic reviews [5, 22] reported comparable implant survivorship and complication rates between KA and MA TKAs, without increasing risk of aseptic loosening at mid‐term follow‐up. Howell et al. [10] demonstrated excellent implant survival for the limb alignments up to 8.5° varus and tibial component alignments up to 7° varus. However, such results can be achieved only if a precise surgical technique is applied, with accurate bone resections of the distal femur and proximal tibia. Indeed, Innocenti et al. [12] demonstrated that changes in implant alignment significantly increase polyethylene and bone stress. Changes in tibial component alignment were found to be more detrimental compared to those of the femoral component [12]. Although some studies proposed reliable anatomic landmarks [30] and advanced inertial‐based cutting guides [25], the risk of misplacement of prosthetic components is high, thereby compromising the force distribution and implant longevity [26]. The biomechanical impact of iKA is still poorly investigated in the literature, and this study represents the first biomechanic evaluation to the best of our knowledge. The behaviour of iKA appears to fall between MA and KA, resembling MA more closely in terms of force distribution (M/L ratio > 1) in case of more pronounced varus deviations, as observed in P2. Further investigation is needed to confirm such findings. However, early results from some clinical reports show encouraging implant survivorship [40].

A further significant finding of this study is the differing kinematic behaviour in the two examined patients, which seems to depend more on the severity of the deformity than on the type of alignment. The two analysed kinematic outputs (AP translation and IE rotation), although differing in magnitude, both show variations across the different alignment strategies. In P1, kinematic changes are minimal, indicating that at low levels of joint deformity, the outcomes associated with different alignment strategies are largely comparable. In contrast, patient P2 exhibits more pronounced differences, particularly in IE rotation. This may have a significant impact on clinical and functional outcomes, since a correlation between patient's satisfaction and change in rotational knee kinematics was previously stated [17]. Liu et al. [23] performed a meta‐analysis on 1112 participants equally distributed in two groups according to the alignment adopted during TKA implantation. Functional outcomes were found superior in patients with KA over MA, as well as the walk distance before discharge was longer in the KA group than in the MA group. In contrast, perioperative clinical outcomes and postoperative complication rates were similar in both groups. However, another systematic review including six studies found no significant difference in any outcomes measured between KA and MA in TKA [33]. A further meta‐analysis of 12 randomised controlled trials was conducted more recently to corroborate such findings [29]. Also in this case, the metanalysis failed to demonstrate any significant improvement in clinical and functional scores within 2 years of follow‐up with KA over MA [29]. It should be stressed that there is persistent confusion in the literature regarding the different types of KA, which are often grouped under a single methodological category [4]. Considering the present study's findings, such generalisation should be avoided, as distinct approaches may yield significantly different outcomes in relation to patient's phenotypes. Literature dealing with outcomes after iKA is more sparce. A retrospective comparative study conducted on two groups of 40 patients reported comparable clinical outcomes at 12‐month follow‐up between iKA and adjusted MA [41]. However, in knees with preoperative varus deformity, iKA yielded significantly better functional outcomes and satisfaction than adjusted MA. The same research group [39] demonstrated in another paper that, at 2 years follow‐up, patients who underwent iKA exhibited gait patterns more closely resembling those of healthy controls compared to patients treated with MA. The restoration of native coronal limb alignment in iKA does not result in increased knee adduction moments, likely due to the preservation of the native obliquity of the TJL.

Although the results of the present study are consistent with existing literature, several limitations must be acknowledged. Only two patients were evaluated, which limits the generalisability of the findings. Future studies should include larger sample sizes and a broader range of morphological conditions to enhance the applicability of the results. Additionally, ligament modelling could be refined to more accurately represent their mechanical behaviour, and the surgical balancing procedure could be simulated to assess its potential impact on the outcomes. Moreover, only one prosthetic design with PS insert was analysed. It is unclear if a different prosthetic design may produce different outcomes; expanding the investigation to include different implant types may help determine whether implant selection influences the results. Lastly, bone stress distribution was not analysed in this study: in the future, integrating bone stress analysis could help identify potential differences in the distribution of mechanical loads across the bone.

## CONCLUSION

This study highlighted the critical interplay between patient‐specific morphology and alignment strategy in TKA. By analysing patients with varying degrees of varus deformity, the analysis showed that similar alignment techniques can lead to distinct biomechanical outcomes. The differences among patients are reduced if MA is used. In contrast, if KA or iKA is adopted, patient‐specific factors may lead to varying results depending on the magnitude of varus deviation. These findings emphasise that alignment should not be evaluated in isolation during preoperative planning. Instead, both alignment philosophy and anatomical deformity must be considered together to achieve optimal functional results.

## AUTHOR CONTRIBUTIONS

**Arcangelo Russo**: Conceptualisation; writing—review and editing; resources; supervision. **Mattia Sisella**: Formal analysis and investigation; writing—original draft preparation; writing—review and editing. **Anna Carrara**: Formal analysis and investigation; writing—original draft preparation. **Nicol Giacoppo**: Formal analysis and investigation; writing—original draft preparation. **Giuseppe Gianluca Costa**: Writing—original draft preparation; writing—review and editing. **Gianluca Zocco**: Writing—original draft preparation; writing—review and editing. **Bernardo Innocenti**: Conceptualisation; methodology; formal analysis and investigation; writing—review and editing; resources; supervision.

## CONFLICT OF INTEREST STATEMENT

The authors declare no conflict of interest.

## ETHICS STATEMENT

The authors have nothing to report.

## Data Availability

Additional data can be provided upon request.
